# Warthin’s Tumor Involved by Monoclonal B-Cell Lymphocytosis: A Case Report

**DOI:** 10.7759/cureus.96758

**Published:** 2025-11-13

**Authors:** Alexie Kindy, Ziang Wang, Robin Gautam, Robert Bennett, Zijian Wang

**Affiliations:** 1 College of Medicine, Northeast Ohio Medical University, Rootstown, USA; 2 Department of Medical Science, 3D Medicines Inc., Rockville, USA; 3 Department of Pathology, Northeast Ohio Medical University/Mercy Health – St. Elizabeth Youngstown Hospital, Youngstown, USA

**Keywords:** chronic lymphocytic leukemia/small lymphocytic lymphoma, monoclonal b cell lymphocytosis (mbl), non-hodgkin's lymphoma, salivary gland tumor, warthin's tumour

## Abstract

Warthin’s tumor is a benign salivary gland neoplasm characterized by oncocytic epithelium and lymphoid stroma. We describe the incidental discovery of a CD5-positive monoclonal B-cell population within a classic Warthin’s tumor of the parotid gland in a 72-year-old woman who presented with a painless, slow-growing left parotid mass. Core needle biopsy and subsequent parotidectomy demonstrated typical histologic features of Warthin’s tumor along with a low-level (8%-9%) CD5- and CD23-positive B-cell population consistent with chronic lymphocytic leukemia (CLL)-type monoclonal B-cell lymphocytosis (MBL). No morphologic evidence of overt lymphoma was present, and both CLL fluorescence in situ hybridization (FISH) testing and PET-CT imaging were unremarkable. The patient remains asymptomatic and is currently monitored without therapy. This case illustrates the occurrence of CLL-type MBL within the lymphoid stroma of a Warthin’s tumor, emphasizing the importance of careful histologic and immunophenotypic evaluation and the need for clearly defined diagnostic criteria for extranodal tissue-based MBL.

## Introduction

Warthin’s tumor, or papillary cystadenoma lymphomatosum, is the second most common benign salivary gland neoplasm and has a strong association with smoking [1]. It typically presents as a painless, slow-growing mass and is most often located in the parotid gland. The tumor is characterized by both epithelial and lymphoid components. While Warthin’s tumor is generally indolent and carries an excellent prognosis, rare cases of malignant transformation have been reported, including carcinoma ex Warthin tumor and primary lymphomas arising within the tumor [2].

Monoclonal B-cell lymphocytosis (MBL) is a lymphoproliferative disorder characterized by a low-level monoclonal B-cell population in asymptomatic individuals, without lymphadenopathy, organomegaly, or other clinical features indicative of overt leukemia or lymphoma. Common sites of detection include the peripheral blood, bone marrow, lymph nodes, and other tissues [3-5]. MBL is considered a potential precursor to chronic lymphocytic leukemia (CLL)/small lymphocytic lymphoma (SLL) and other indolent B-cell lymphomas. The most common immunophenotype of MBL is the CLL-type, characterized by expression of CD19, CD5, CD23, and dim CD20. A subset of cases demonstrates an atypical CLL-like immunophenotype, with expression of CD19, CD5, bright CD20, and variable CD23 expression. A minority of cases exhibit a non-CLL immunophenotype, lacking CD5 expression [3].

Diagnosing primary lymphoproliferative disorders arising within a Warthin tumor presents a unique diagnostic challenge. In routine surgical pathology practice, attention is often directed toward evaluating the epithelial component of the tumor, which constitutes the defining feature of Warthin tumor, whereas the accompanying lymphoid stroma is frequently regarded as reactive in nature and therefore receives less detailed scrutiny. However, the lymphoid component of a Warthin tumor can display a broad morphologic spectrum. It may or may not contain well-formed lymphoid follicles, reactive germinal centers, or paracortical regions rich in histiocytes, reflecting varying degrees of immune reactivity. When overt morphologic abnormalities such as follicular expansion, increased numbers of large atypical lymphocytes, or intraepithelial lymphoid infiltration are observed, pathologists typically initiate a lymphoma workup following standard protocols, including immunohistochemical studies. Nonetheless, subtle or early-stage lymphoproliferative processes may escape recognition, particularly when they lack overt architectural distortion or cytologic atypia. Consequently, primary lymphoproliferative disorders within Warthin tumors, especially indolent entities such as MBL, may be underdiagnosed or completely overlooked. Recognition of such early clonal proliferations is important, as they may represent the earliest identifiable stages in the spectrum of B-cell lymphoproliferative neoplasia.

In this report, we present the first documented case of a Warthin tumor partially involved by CLL-type MBL. This case suggests the possibility of primary lymphomatous transformation within the lymphoid stroma, rather than secondary involvement from systemic lymphoma. Although MBL is typically defined in peripheral blood, bone marrow, and lymph nodes, criteria for identifying tissue-based or extranodal MBL, particularly within benign tumors containing lymphoid elements, remain poorly established, creating a diagnostic challenge. This case highlights the importance of careful morphologic assessment and immunophenotypic analysis of the lymphoid stroma, even when overt atypia is absent, and expands current understanding of early B-cell clonal proliferations that may arise in salivary gland neoplasms.

## Case presentation

A 72-year-old female patient presented with a progressively enlarging left parotid mass, first noted approximately four months prior to presentation. The patient had no pain, drainage, hoarseness, weight loss, dysphagia, or dyspnea. Complete blood count (CBC) was in the normal range, and past medical/surgical history was non-significant. An ultrasound was performed, detecting an indeterminate 2.2-cm complex cyst of the left parotid. The patient was referred to the radiology department for a biopsy. A core needle biopsy was performed, and a representative section was sent for flow cytometry. The intraoperative rationale for this decision remains unclear.

Microscopic examination of the biopsied specimen revealed a papillary and cystic lesion composed of epithelial and lymphoid components. The epithelial lining consisted of a double layer of granular oncocytic cells, while the lymphoid component contained a mixture of small, mature lymphocytes with scattered primary and secondary follicles. These findings were consistent with a classic Warthin’s tumor.

However, flow cytometry identified a monoclonal B cell population (9% of total cells), negative for kappa and lambda light chains, co-expressing CD19, CD5, dim CD20, and CD23, while negative for CD10 and CD38. These findings suggested an underlying B‑lymphoproliferative disorder.

The patient subsequently underwent a left parotidectomy. Gross examination revealed a well-circumscribed 1.8 × 1.7 × 1.5 cm mass within otherwise unremarkable parotid tissue. Representative tissue was retained for flow cytometry, and multiple sections were submitted for permanent histologic examination. Although the lesion appeared benign on gross inspection, the possibility of early lymphoproliferative involvement or reactive lymphoid hyperplasia could not be excluded, underscoring the importance of comprehensive histopathologic and immunophenotypic evaluation.

**Figure 1:**
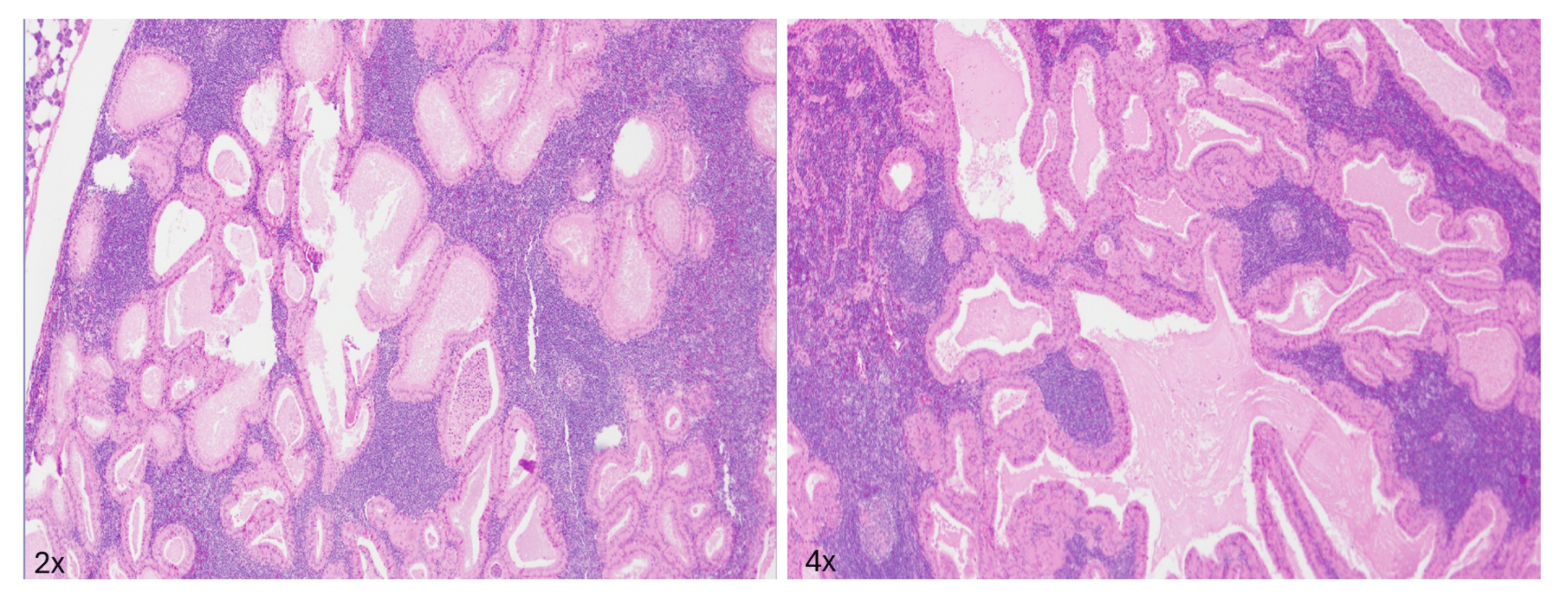
Left parotidectomy, H&E 2x and 4x A well-circumscribed papillary and cystic mass composed of oncocytic epithelial and lymphoid elements, consistent with Warthin’s tumor.

Histologic examination demonstrated a well-circumscribed papillary and cystic mass composed of oncocytic epithelial and lymphoid elements, consistent with Warthin’s tumor (Figure 1). The lymphoid component consisted of mixed small, mature lymphocytes with germinal center-containing follicles. No atypical lymphoid cells or proliferation centers were identified.

**Figure 2:**
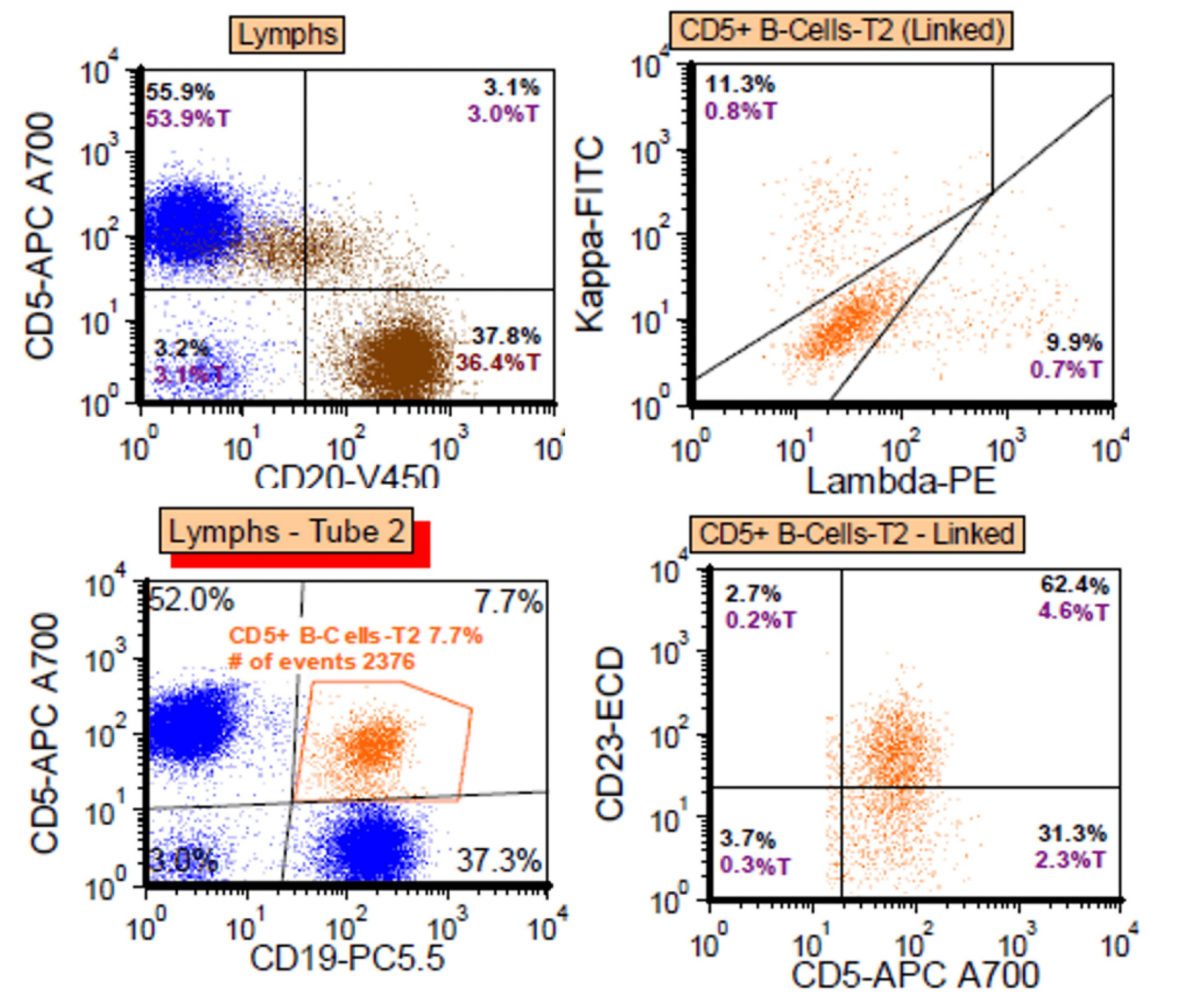
Flow cytometry study of the left parotidectomy Kappa/lambda negative monoclonal B-cells (8% of lymphoid cells) express: CD19 mod, CD20 dim, CD5 mod, and CD23 mod.

Repeated flow cytometry identified a subset of monoclonal B cells (8% of lymphoid cells), demonstrating a similar immunophenotype to the previous study, with co-expression of CD19 (moderate), CD20 (dim), CD5 (moderate), CD10 (negative), CD11c (dim), CD23 (moderate), CD38 (negative), HLA-DR (moderate), and kappa/lambda negativity (Figure 2).

**Figure 3:**
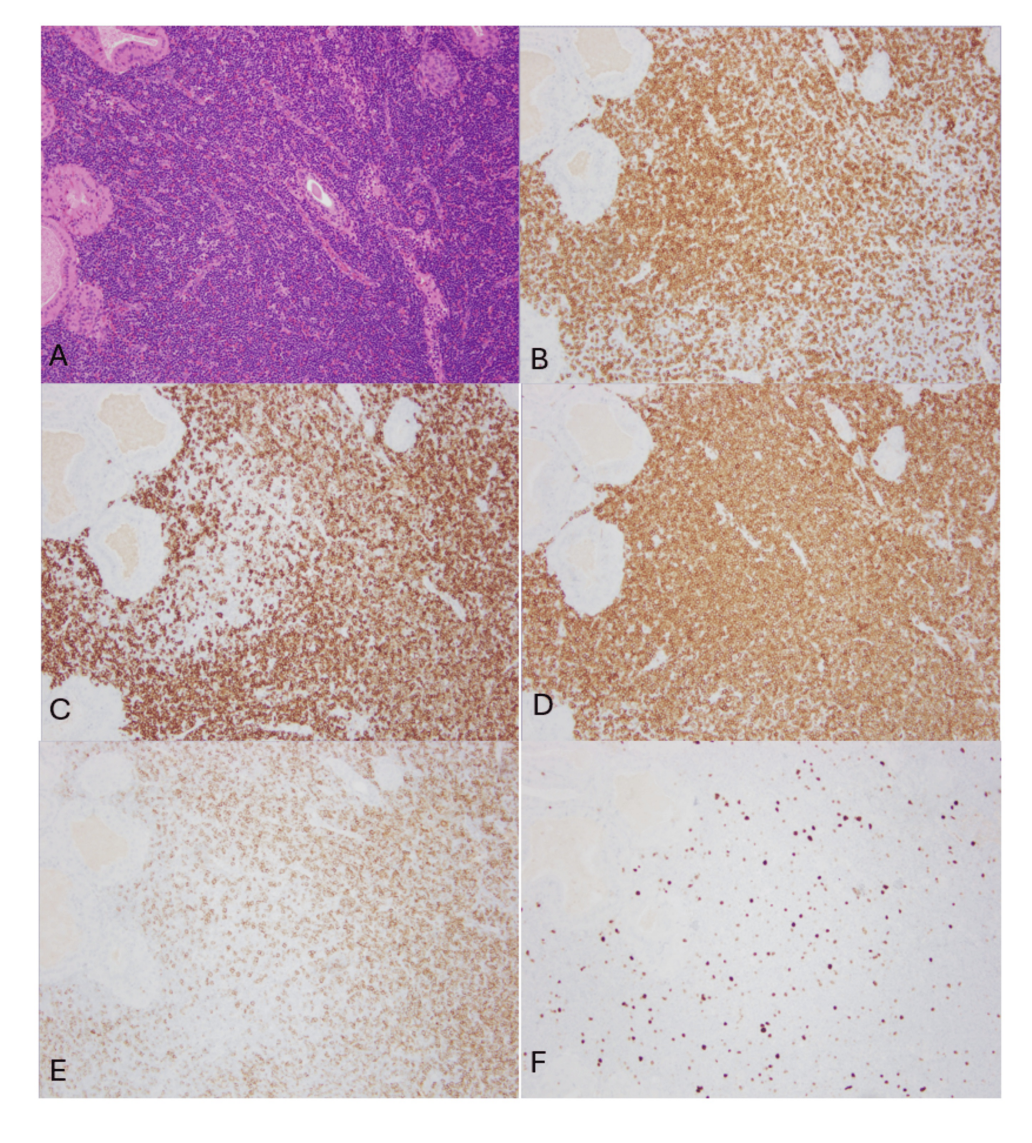
Left parotidectomy, non-follicular area A. (H&E, 4x) The lymphoid component consisted of mixed cells showing small lymphocytes, histiocytes, and occasional immunoblastic cells, with patent sinuses. B. CD3-positive T lymphocytes. C. CD20-positive B lymphocytes. D. CD5-positive cells outnumbered CD3-positive cells, indicating the presence of CD5-positive B lymphocytes in the background. E. Subsets of B lymphocytes also expressed CD23.  F. Ki-67 staining demonstrated a low proliferative index (<10%).

A detailed morphologic and immunohistochemical evaluation was then performed. Microscopic examination of the non-follicular areas (Figure 3) showed small lymphocytes, histiocytes, and occasional immunoblasts, with patent sinuses. Immunohistochemistry revealed an admixture of CD3-positive T lymphocytes and CD20-positive B lymphocytes. CD5-positive cells outnumbered CD3-positive cells, indicating the presence of CD5-positive B lymphocytes in the background. Subsets of B lymphocytes also expressed CD5 and CD23. Ki-67 staining demonstrated a low proliferative index (5%-10%).

**Figure 4:**
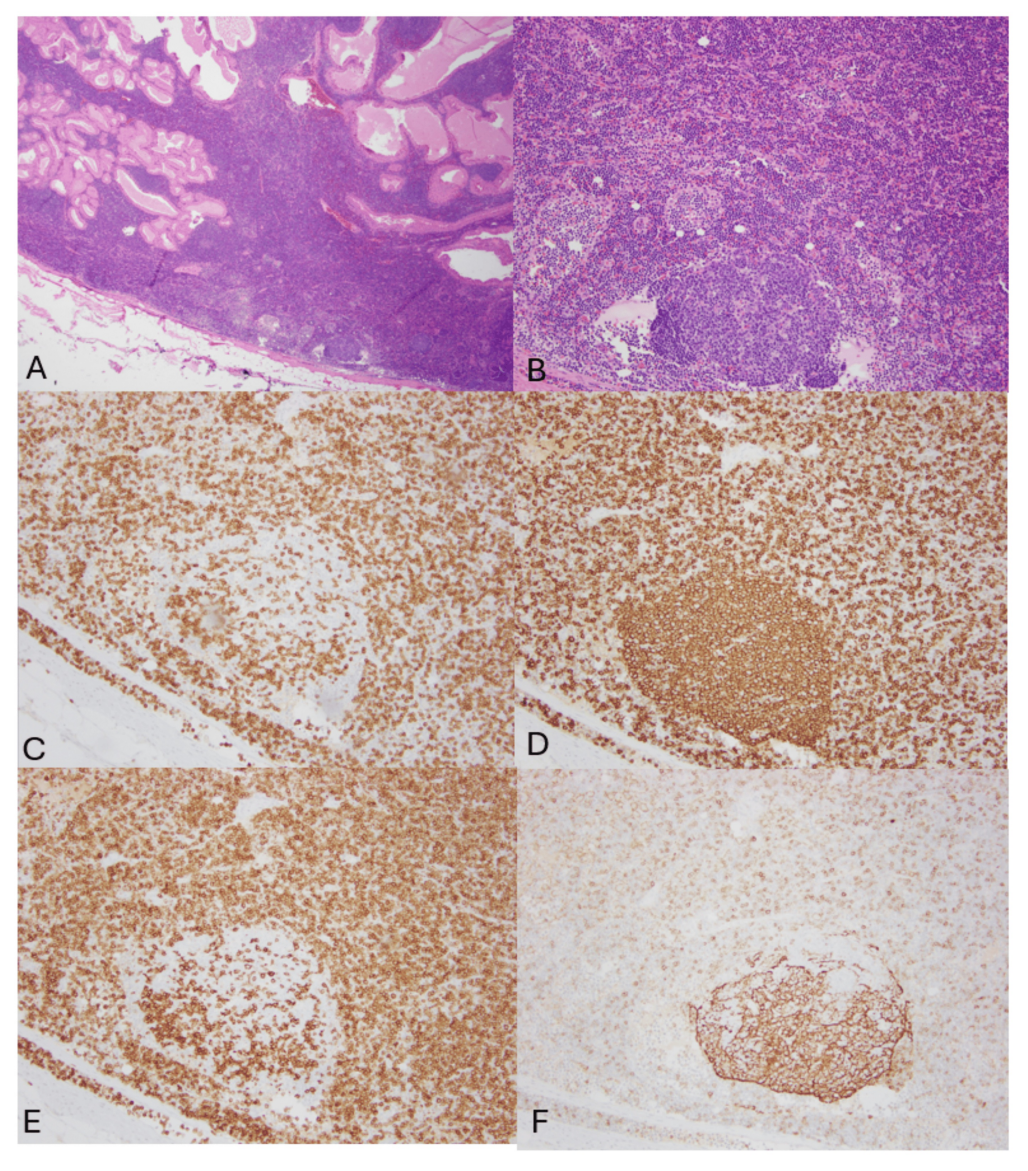
Left parotidectomy, follicular area A & B. (H&E, 2x and 10x) In follicular-rich areas, primary and secondary follicles were observed. C. CD3-positive T lymphocytes, primarily in the paracortex, also highlight T follicular helper cells within follicles. D. CD20-positive B lymphocytes, distributed across follicles and perifollicular areas. E. CD5-positive cells outnumbered CD3-positive cells, indicating the presence of CD5-positive B lymphocytes in the background. F. CD23 highlighted follicular dendritic meshworks within follicles. Subsets of B lymphocytes also expressed CD23.

**Figure 5:**
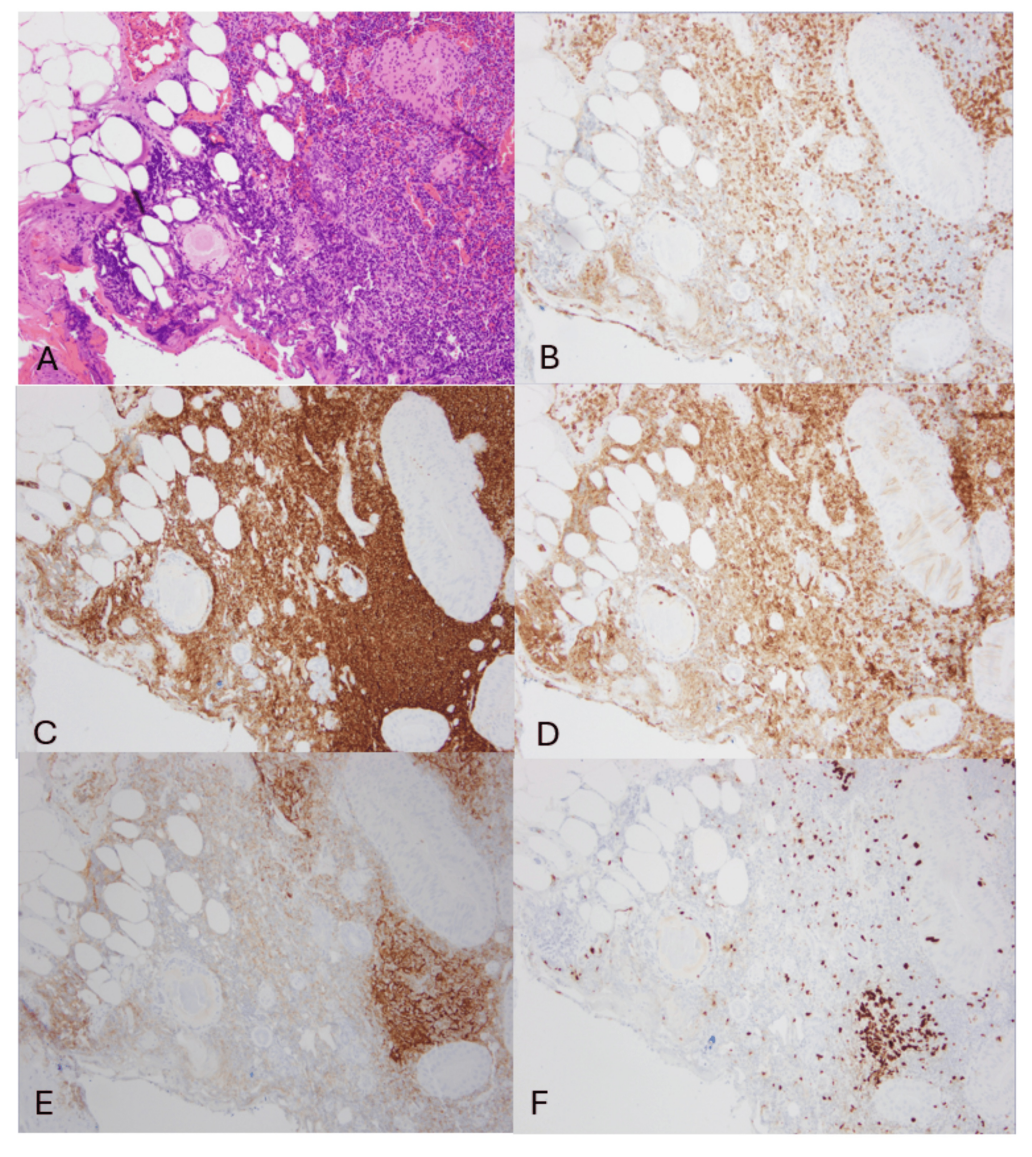
Left parotidectomy, B-cell-rich area A. (H&E, 10x) The B-cell-rich area exhibits possible lymphoid infiltration; however, a tangential cut artifact is favored. B. CD3-positive T lymphocytes. C. Markedly increased CD20-positive B lymphocytes. D. CD5-positive cells outnumbered CD3-positive cells, indicating the presence of CD5-positive B lymphocytes in the background. E. CD23 highlighted follicular dendritic meshworks within follicles. Subsets of B lymphocytes also expressed CD23.  F. Ki-67 highlights approximately 40% of cells within germinal centers with polarity. Less than 5% outside follicles are noted.

In follicular-rich areas (Figure 4), both primary and secondary follicles were identified. These regions contained a mixture of lymphoid cells, including CD3-positive T lymphocytes and CD20-positive B lymphocytes. B lymphocytes were present within the follicles and extended into the perifollicular zones, with subsets showing expression of CD5 and CD23. CD23 also delineated follicular dendritic cell meshworks within the follicles. In B-cell-rich areas (Figure 5), only a subset of B lymphocytes expressed CD5 and CD23. Cyclin D1 staining was negative in the lymphoid population. These immunohistochemical findings were consistent with the results of flow cytometry. Overall, the morphologic and immunophenotypic features were most consistent with Warthin’s tumor with focal involvement by a low-grade CD5-positive B-cell lymphoproliferative disorder, favoring MBL, CLL-type. However, the possibility of an overt lymphoma at another site could not be entirely excluded.

Further evaluations included a PET-CT scan and cytogenetic studies using a CLL fluorescence in situ hybridization (FISH) panel. The PET-CT revealed only mild heterogeneous activity in the left parotid gland, consistent with postsurgical changes, with no evidence of metabolically active lymphadenopathy. The CLL FISH panel showed no cytogenetic abnormalities. Overall, the findings were suggestive of a diagnosis of MLB of CLL-type. The patient is being managed with close follow-up without chemotherapy.

## Discussion

Warthin’s tumor is the second most common salivary gland neoplasm and is typically composed of both proliferative epithelial and lymphoid components [1]. The origin of the lymphoid stroma in Warthin’s tumor has been the subject of debate for many years [6]. The most widely accepted hypothesis suggests that Warthin’s tumor is a neoplasm that develops in the heterotopic salivary gland ducts within or adjacent to parotid lymph nodes [7]. Malignant transformation of Warthin’s tumor is rare. While carcinomatous transformation is well documented, lymphoid transformation is exceedingly uncommon [2]. Our review of the 33 reported cases of coexisting Warthin tumor and non-Hodgkin lymphoma, as summarized in Table 1, shows that the most frequently reported subtype is follicular lymphoma (15 cases) [8]. Other reported lymphoproliferative disorders include one case of follicular lymphoma in situ [8], seven cases of diffuse large B-cell lymphoma [9-11], six cases of small lymphocytic lymphoma (SLL) [12-14], two cases of mantle cell lymphoma [15, 16], and a single case of mucosa-associated lymphoid tissue lymphoma [17] and nodular peripheral T-cell lymphoma [18]. To our knowledge, the presence of MBL within a Warthin’s tumor has not been previously reported. 

**Table 1:** Summary of cases of non-Hodgkin lymphoma arising in Warthin tumor MALT: mucosa-associated lymphoid tissue

Lymphoma Type	Number of Cases	References
Follicular lymphoma	15	[8]
In situ follicular neoplasia	1	[8]
Diffuse large B-cell lymphoma	7	[9–11]
Small lymphocytic lymphoma	6	[12-14]
Mantle-cell lymphoma	2	[15, 16]
MALT-type lymphoma	1	[17]
Nodular peripheral T-cell lymphoma	1	[18]

Although MBL is traditionally defined on the basis of peripheral blood findings, pathologists occasionally encounter cases in which a monotypic B-cell population is detected in tissue biopsies by flow cytometry, without accompanying morphologic evidence of lymphoma. This phenomenon, referred to here as “tissue MBL,” has not been well characterized, particularly in comparison to classic blood-based MBL. In a study by Gibson et al. [5], 36 patients with extramedullary tissue biopsies showing either SLL or monotypic B cells with a CLL immunophenotype, who also had peripheral blood MBL (<5 × 10³/μL), were evaluated. Proliferation centers and lymph nodes larger than 1.5 cm were associated with disease progression or the need for therapy, whereas small lymph nodes containing CLL-phenotype clones without proliferation centers were suggested to represent the tissue counterpart of MBL. Whether tissue MBL can exist independently of peripheral blood involvement, and how it should be defined diagnostically, remain important unresolved questions.

The diagnosis of MBL in our case, rather than CLL/SLL, is supported by clinical and pathological findings. Clinically, the patient is hematologically asymptomatic, with a normal CBC and no evidence of lymphadenopathy, as supported by PET-CT findings. The neoplasm, measuring 2.2 cm, slightly exceeds the 1.5 cm threshold sometimes used to help differentiate between MBL and CLL in extra-nodal sites [3, 7]. However, the size remains well within the typical 1-8 cm size range for Warthin’s tumors. These findings are consistent with a localized process rather than a systemic disease process. Histologically, the lymphoid element of Warthin’s tumor appears benign, preserving its normal architecture with both primary and secondary follicles. No proliferation centers or atypical lymphoid cells are identified on morphologic review, features that are characteristic of CLL. Finally, cytogenetic testing using a CLL FISH panel shows no detectable abnormalities. Together, these features point toward a diagnosis of MBL, an incidental, low-level monoclonal process, rather than overt leukemia/lymphoma.

## Conclusions

Collectively, this case highlights the possibility of primary lymphomatous transformation originating in the lymphoid stroma of a Warthin’s tumor, rather than secondary involvement by systemic lymphoma or metastatic disease. Current definitions of MBL encompass its detection in peripheral blood, bone marrow, and lymph nodes. Diagnostic criteria for identifying tissue MBL at extra-nodal sites, particularly within benign tumors containing lymphoid elements, remain undefined, making such diagnoses especially challenging. This underscores the importance of careful morphologic evaluation and the use of immunohistochemical studies in suspected cases in order to detect subtle lymphomatous involvement. Our case demonstrates a critical gap in the current understanding of MBL’s manifestation in soft tissue and emphasizes the need for further investigation to elucidate its clinical relevance and to establish clear diagnostic guidelines in such settings.
